# Berberine Protects Against Simulated Ischemia/Reperfusion Injury-Induced H9C2 Cardiomyocytes Apoptosis *In Vitro* and Myocardial Ischemia/Reperfusion-Induced Apoptosis *In Vivo* by Regulating the Mitophagy-Mediated HIF-1α/BNIP3 Pathway

**DOI:** 10.3389/fphar.2020.00367

**Published:** 2020-03-27

**Authors:** Na Zhu, Jiang Li, Yongli Li, Yuwei Zhang, Qiubo Du, Peiyuan Hao, Jinying Li, Xueming Cao, Li Li

**Affiliations:** ^1^Department of Health Management, Henan Provincial People's Hospital, People's Hospital of Zhengzhou University, School of Clinical Medicine, Henan University, Zhengzhou, China; ^2^Henan Provincial Research Center of Natural Medicine Extraction and Medical Technology Application Engineering, Zhengzhou Railway Vocational Technical College, Zhengzhou, China; ^3^Medical Genetic Institute of Henan Province, Henan Provincial People's Hospital, Zhengzhou University People's Hospital, School of Clinical Medicine, Henan University, Zhengzhou, China; ^4^Department of Cardiology, Henan Provincial Key Lab for Control of Coronary Heart Disease, Henan Provincial People's Hospital, Central China Fuwai Hospital, Zhengzhou University People's Hospital, School of Clinical Medicine, Henan University, Zhengzhou, China; ^5^Department of Scientific Research and Discipline Construction, Henan Provincial People's Hospital, Zhengzhou University People's Hospital, Henan University People's Hospital, Zhengzhou, China

**Keywords:** berberine, myocardial ischemia injury, myocardial reperfusion injury, mitophagy, HIF-1α/BNIP3 pathway

## Abstract

Berberine (BBR) has a variety of pharmacological activities and is widely used in Asian countries. However, the clinical application of BBR still lacks scientific basis, what protective mechanism of BBR against myocardial ischemia-reperfusion injury (MIRI). *In vitro* experiments, BBR pretreatment regulated autophagy-related protein expression, induced cell proliferation and autophagosome formation, and reduced the mitochondrial membrane potential (ΔΨm) increase in H9C2 cells. *In vivo* experiments, BBR reduced the myocardial infarct size, decreased cardiomyocyte apoptosis, and markedly decreased myocardial enzyme (CK-MB, LDH, and AST) activity-induced I/R. In addition, upon BNIP3 knockdown, the regulatory effects of BBR on the above indicators were weakened both in H9C2 cells and *in vivo*. Luciferase reporter and ChIP assays indicated that BBR mediated BNIP3 expression by enhancing the binding of HIF-1α to the BNIP3 promoter. BBR protects against myocardial I/R injury by inducing cardiomyocytes proliferation, inhibiting cardiomyocytes apoptosis, and inducing the mitophagy-mediated HIF-1α/BNIP3 pathway. Thus, BBR may serve as a novel therapeutic drug for myocardial I/R injury.

## Introduction

Coronary heart disease (CHD) has become the leading cause of human death, accounting for 13.2% of the top 10 causes (Lang et al., 2017). The most effective therapeutic intervention for CHD is timely and effective myocardial reperfusion. However, the process of myocardial reperfusion can itself induce further cardiomyocyte death and result in myocardial ischemia-reperfusion injury (MIRI) (Britto et al., 2018). The molecular and cellular events underlying MIRI are complex due to the confluence of divergent biological pathways (Turer and Hill, 2010). Currently, there remains no effective clinical therapy for preventing myocardial reperfusion injury (Hausenloy and Yellon, 2013).

MIRI research has shown that mitochondrial autophagy can eliminate damaged mitochondria and maintain intracellular environment homeostasis and cardiomyocyte function in MIRI and that mitochondrial autophagy regulation is an effective method for protecting against MIRI (Dong et al., 2010; Yang et al., 2010). Decreasing expression of the proapoptotic protein BNIP3 can promote mitochondrial autophagy and accelerate damaged mitochondria removal (Hamacherbrady et al., 2007). Wu et al. indicated that trimetazidine inhibited excessive I/R-induced autophagy and apoptosis partly by activating the AKT/mTOR pathway, and this ultimately protected against MIRI (Wu et al., 2018). Therefore, exploring the regulatory mechanism of mitochondrial autophagy in MIRI and identifying substances that can regulate mitochondrial autophagy are crucial for the treatment and prevention of CHD.

Berberine (BBR) is an alkaloid extracted from medicinal plants such as Coptis chinensis and *barberry*. It has a variety of pharmacological effects and has been used for the treatment of various diseases (Liu et al., 2006; Imanshahidi and Hosseinzadeh, 2008). In cell and animal experiments, BBR has antiinflammatory (Liu et al., 2004) and antiapoptotic effects (Zhang and Ney, 2009). In clinical studies, BBR has been shown to be a potent oral hypoglycemic agent with beneficial effects on lipid metabolism in type 2 diabetes mellitus patients (Yin et al., 2008). Importantly, the effects of BBR on MIRI have attracted the attention of researchers. Huang et al. found that BBR treatment significantly enhanced H/R-induced cell viability and reduced I/R-induced myocardial infarct size and cellular autophagy levels (Huang et al., 2015). In addition, BBR decreased CK-MB, LDH and cTnI serum levels, attenuated myocardial apoptosis and improved mitochondrial dysfunction (Wang et al., 2015). Moreover, BBR exhibited neuroprotective effects *via* promoting autophagy and decreasing anoxia-induced apoptosis (Zhang et al., 2016). Based on these studies, BBR can clearly protect the heart from ischemia/reperfusion (I/R) injury, but the specific protective mechanism is unclear, and further research is needed.

Previous research by our team found that BBR attenuates mitochondrial dysfunction by inducing autophagic flux in myocardial H/R injury (Cao, 2014). In addition, as a target gene directly regulated by HIF-1, BNIP3 is an important signaling molecule in hypoxia-induced mitochondrial autophagy and plays an important role in ischemia- and ischemia-reperfusion-induced autophagy (Swiderek et al., 2013; Chen-Scarabelli et al., 2014). Therefore, we hypothesized that BBR might regulate mitochondrial autophagy *via* the HIF-1α/BNIP3 signaling pathway. In this study, we firstly established hypoxia/reoxygenation (H/R) and I/R models and then performed BBR pretreatment and siRNA/shRNA interference experiments to ultimately reveal the underlying therapeutic mechanisms of BBR on AMI.

## Materials and Methods

### H9C2 Cell Experiment

H9C2 cells were obtained from the American Type Culture Collection (http://www.atcc.org/, #CRL-1446). The cells were cultured in Dulbecco's modified Eagle's medium (DMEM) supplemented with 10% fetal bovine serum (FBS) at 37°C in a 5% CO_2_ humidified atmosphere. BBR chloride was obtained from Sigma-Aldrich (#B3251) and diluted in 10% methanol as a stock solution. The final concentration of methanol never exceeded 0.1% in the control or treated cells/rats.

H9C2 cells were divided into the following five groups: (1) untreated control group (control); (2) H/R group: H9C2 cells were exposed to 4 h of hypoxia followed by 3 h of reoxygenation; (3) BBR+H/R group: H9C2 cells were pretreated with BBR for 3 h (50 μM) prior to H/R; (4) siNC+BBR+H/R group: H9C2 cells were transfected with siNC and then treated with BBR for 3 h (50 μM) prior to H/R; and (5) siBNIP3+BBR+H/R group: H9C2 cells were transfected with siBNIP3 and then treated with BBR for 3 h (50 μM) prior to H/R.

The siBNIP3 and siNC groups were transfected respectively with small interfering RNA (siRNA) against BNIP3 (siBNIP3) to knock down BNIP3 expression and with negative control siRNA (siNC) using Exfect Transfection Reagent (Vazyme Biotech, Nanjing, China) according to the manufacturer's instructions. The siBNIP3 and siNC oligonucleotide duplex sequences are listed in **Supplemental Table 1**. The siRNAs were purchased from Shanghai GenePharma Co., Ltd. At 48 h posttransfection (hpt), the cells were processed for BBR coculture (3 h, 50 μM) and H/R treatment.

At 51-h hpt, the cells were subjected to H/R. Hypoxia was created by incubating the cells in an airtight Plexiglas chamber with 5% CO_2_/95% N_2_ at 37°C using a GasPak Plus System (BD, USA). After 4 h of hypoxia, the cells were exposed to a normoxic atmosphere (reoxygenation) by replacing the medium with 5% CO2/95% air for 3 h. Then, the cells were collected and processed for further analysis.

### Cell Proliferation Assay

After H/R treatment, the cells were collected, seeded in triplicate in 96-well plates (5×10^3^ cells per well) and cultured for 0, 12, 24, or 48 h. After this culture, added 10 μl of Cell Counting Kit 8 (CCK-8) working reagent (Beyotime, China) to the medium, and incubated for another 1 h. Cell proliferation was assessed at 0, 12, 24, and 36 h by measuring the absorbance at 450 nm using a microplate reader (680 Microplate Reader; Bio-Rad Laboratories, Hercules, CA, USA). The assays were repeated three times.

### Western Blotting

After H/R treatment, cells were lysed with NP40 lysis buffer (Beyotime, P0013F, China). Total protein was quantified with a BCA Protein Assay Kit (Beyotime, P0010, China), resolved by 12% sodium dodecyl sulfate-polyacrylamide gel electrophoresis and then transferred onto polyvinylidene difluoride membranes (Millipore, Germany). After blocking with 5% nonfat milk at 37°C for 1 h, the membranes were probed with anti-LC3 (Abcam, ab48394, USA), anti-P62 (Abcam, ab56416 USA), anti-NIX (Abcam, ab8399, USA), BNIP3 (Abcam, ab10433, USA), or anti-GAPDH (Abcam, ab181602, USA) antibodies at 37°C for 2 h. Secondary antibodies were HRP-conjugated against rat (Abcam, ab6734, USA). Protein levels were normalized to that of GAPDH. Western blotting densitometry values were quantified with ImageJ software.

### RNA Preparation and Real-Time PCR

Total RNA was extracted from cells using RNA Isolater Total RNA Extraction Reagent (R401-01, Vazyme). RNA (500 ng) from each sample was reverse-transcribed into cDNA using a PrimeScript RT reagent kit (Takara, China). A 7500 real-time PCR (RT-PCR) system (Applied Biosystems) with AceQ qPCR SYBR Green Master Mix (Q111-02, Vazyme) performed RT-PCR. RT-PCR primers are presented in **Supplemental Table 2**. The obtained data were normalized to GAPDH expression levels and calculated using the 2^-ΔΔCq^ formula for each sample.

### Mitochondrial Membrane Potential (ΔΨm)

The ΔΨm in H9C2 cells was assessed using rhodamine-123 (R123) as the fluorescent probe by flow cytometry. After H/R treatment, incubated the H9C2 cells with R123 (0.01 mg/ml) for 30 min at 37°C and then subjected to flow cytometry at an emission wavelength of 582 nm. The data were analyzed using Cell Quest software.

### Transmission Electron Microscopy Assay

H9C2 cells were double-fixed in 1.5% glutaraldehyde and 1% osmium tetroxide. Heart tissues were perfused with 4% paraformaldehyde and then double-fixed in 1.5% glutaraldehyde and 1% osmium tetroxide. After dehydration in acetone, H9C2 cells and hearts were treated with propylene oxide and embedded in EMbed 812/Araldite. Ultra-thin slices were prepared using a LEICA UC6 slicer and then double-stained with uranyl acetate and lead citrate. Finally, observed the myocardium ultrastructure and autophagy formation by transmission electron microscopy (TEM) (Philips, Amsterdam, Dutch).

### Plasmid Constructs and Dual-Luciferase Assay

Rat cDNA encoding HIF-1α was amplified from H9C2 cell mRNA by RT-PCR and cloned into the pCMV-flag-N expression vector (Clontech). The rat BNIP3 promoter was amplified from H9C2 cells by PCR and cloned into the pGL3-basic plasmid (Promega, Madison, WI) to construct the BNIP3 luciferase reporter plasmid. All constructs were confirmed by DNA sequencing.

H9C2 cells were cotransfected with a mixture of the BNIP3 luciferase reporter plasmid and pCMV-flag-HIF-1α plasmid. Total amounts of plasmid DNA were equalized using an empty control vector. After transfection for 24 h, H9C2 cells were divided into the following three groups: control, H/R, and BBR+H/R groups. After H/R treatment, the luciferase activities were measured with a Dual-Luciferase Reporter Assay System (Promega) according to the manufacturer's instructions. The data were normalized for transfection efficiency by dividing the firefly luciferase activity by the Renilla luciferase activity.

### Chromatin Immunoprecipitation

H9C2 cells were divided into the following five groups: control group, H/R group, BBR+H/R group, siNC+BBR+H/R group, and siHIF-1α+BBR+H/R group. The siHIF-1α and siNC groups were transfected with siHIF-1α and siNC. At 48-h hpt, the cells were processed for BBR coculture (3 h, 50 μM) and H/R treatment. Then, the cells were collected and processed for chromatin immunoprecipitation (ChIP) analysis.

The ChIP assay was performed using a Magna ChIP kit (Millipore, USA). Briefly, H9C2 cells were treated with 1% formaldehyde to crosslink proteins and DNA. After lysis, the lysate was incubated with an anti-HIF-1α antibody (Abcam, ab1, USA) at 4°C overnight. The next day, protein A-Sepharose beads (Pharmacia Biotech) were added and incubated for 1 h at 4°C to collect the immunoprecipitated complexes; then, the immunoprecipitated complexes were eluted. Next, the crosslinks were reversed by heating the sample, and DNA was extracted using the phenol-chloroform method. The DNA was then ethanol precipitated and resuspended in water. Finally, quantitative RT-PCR was performed using AceQ qPCR SYBR Green Master Mix (Q111-02, Vazyme).

### *In Vivo* Experiment

Ten-week-old Wistar rats (180~200 g, n = 25) were purchased from the Animal Research Centre of Zhengzhou University (Zhengzhou, China). The rats had free access to a normal diet and water and were housed at 25°C ± 2°C under a 12-h light/dark cycle. The experimental studies were approved by the Institutional Animal Care and Use Committee of Henan Provincial People's Hospital.

Rats were divided into the following five groups: (1) sham control group (sham); (2) I/R group: rats were exposed to 30 min of ischemia followed by 120 min of reperfusion; (3) BBR+I/R group: rats were pretreated with BBR for 3 days (300 mg/kg) prior to I/R; (4) shNC+BBR+H/R group: rats were transfected with Ad-shNC and then treated with BBR for 3 days (300 mg/kg) prior to I/R; and (5) shBNIP3+BBR+H/R group: rats were transfected with Ad-BNIP3 and then treated with BBR for 3 days (300 mg/kg) prior to I/R.

Adenoviral vectors were synthesized by Shanghai GenePharma Co., Ltd. AAV9 adenoviral vectors expressing rat BNIP3 shRNA (Ad-shBNIP3) were used to inhibit BNIP3 expression, while AAV9 adenoviral vectors expressing rat scrambled shRNA (Ad-shNC) were used as negative controls. For infection with Ad-BNIP3 or Ad-shNC, injected 50 µl of viral particles at a concentration of 4 × 10^8^ transfection units into the tail vein of each rat using a microinjector. After 48 h of infection, the rats were orally administered BBR (300 mg/kg, once a day for 3 consecutive days) prior to H/R exposure.

Preparation of the MIRI model: The MIRI model was prepared by permanent ligation of the left anterior descending coronary artery with a 6/0 braided silk suture (Zeng et al., 2018). First, the rats were anesthetized using 400 mg/kg 10% chloral hydrate and intubated. Then, a left thoracotomy was performed at the fourth intercostal space, and an artificial ventilator was used to provide breathing assistance during the experiments. A standard lead II electrocardiogram (ECG) was used to monitor cardiac changes. Successful modeling was indicated when the ligated area of the heart became white, the pulse weakened, and the ECG ST segment arched significantly upward. After successful modeling, the rats were euthanized by exsanguination (total blood collection) under deep diethyl ether anesthesia. The rats that had undergone only thoracotomy served as the sham group. The hearts were excised for 2,3,5-triphenyl tetrazolium (TTC) and terminal deoxynucleotidyl transferase-mediated dUTP nick-end labeling (TUNEL) staining, and the blood was collected and centrifuged for creatine kinase MB isoenzyme (CK-MB), lactate dehydrogenase (LDH) and aspartate aminotransferase (AST) measurements. The mRNA and protein expression levels of BNIP3, LC3, NIX, and P62 were detected by RT-PCR and Western blotting.

### ELISA

The concentrations of CK-MB, LDH, and AST were measured using ELISA kits. CK-MB was examined using a Creatine Kinase Activity Assay Kit (Sigma-Aldrich, #MAK116), LDH was assessed using an LDH ELISA Kit (Sigma-Aldrich, #MAK066-1KT), and AST was measured using an AST Activity Assay Kit (Sigma-Aldrich, #MAK055). All assays were performed by following the manufacturer's instructions exactly.

### TTC Staining

Briefly, cut the hearts into 4-mm slices and immediately immersed in a 1% TTC solution (Sigma Chemical Co.) in phosphate buffer (PBS, pH 7.4) for 20 min at room temperature. After being washed three times, the slices were photographed, and the infarct area was compared with the total area using digital planimetry software (Image-Pro Plus 6.0).

### TUNEL Assay

Partial hearts were fixed in 4% paraformaldehyde for 60 min, permeabilized with 0.1% Triton X-100 for 10 min, and finally washed three times with PBS. The procedure was performed using an In Situ Cell Death Detection Kit (Roche, No: 11684817910, Germany), and counterstaining was performed using DAB. Images were photographed with a fluorescence microscope (Olympus, CX71, Japan) at 400× magnification. TUNEL index = (TUNEL-positive cells/DAB-labeled cells) ×100.

### Statistical Analysis

The data were statistically analyzed and graphed using GraphPad Prism 5 (GraphPad Software, USA). All results are presented as the mean values ± standard deviations. Statistically significant differences between groups were determined by Student's *t*-test. Multiple comparisons were made among ≥3 groups using 1-way ANOVA followed by the Bonferroni *post hoc* test. A nonparametric Mann-Whitney U test was used if the data were not normally distributed. ^*^P < 0.05 and ^**^P < 0.01 were considered statistically significant.

## Results

### BBR Regulates BNIP3-Mediated Mitochondrial Autophagy to Attenuate H/R Injury in H9C2 Cells

In the H/R H9C2 cell and MIRI rat models, RT-PCR and Western blotting indicated that BNIP3 expression levels were significantly increased compared with those in cells and rats without H/R or I/R treatment (*P* < 0.05). Additionally, BBR coculture could increase the expression levels of BNIP3 (**Supplemental Figures 1A, B**, and **Supplemental Figures 2A, B**). Then, we performed a BNIP3 silencing transfection experiment in H9C2 cells and rats. RT-PCR and Western blotting verified that the transfection efficiency was high and that BNIP3 silencing was achieved (**Supplemental Figures 1C, D** and **Supplemental Figures 2C, D**).

**Figure 1:**
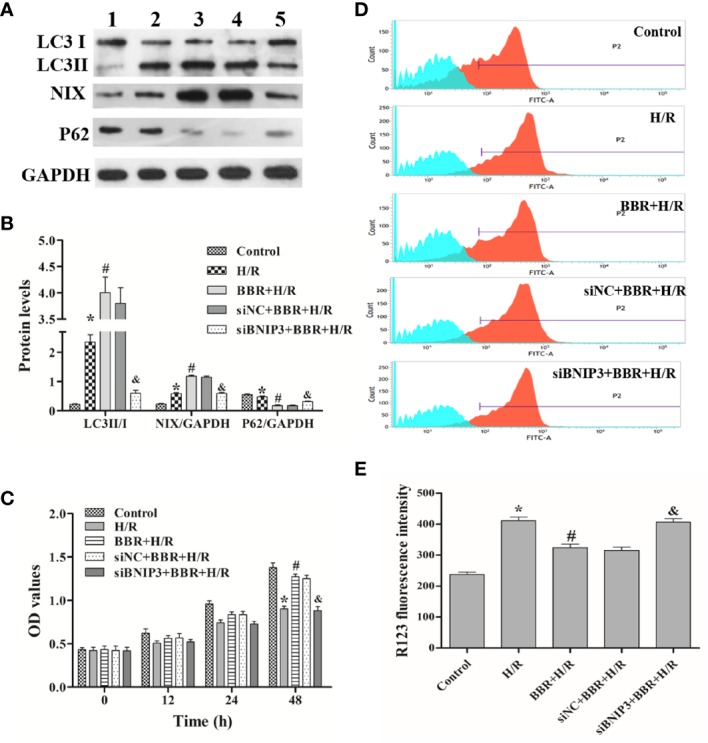
Berberine (BBR) regulates BNIP3-mediated mitochondrial autophagy to attenuate hypoxia/reoxygenation (H/R) injury in H9C2 cells. After transfection with small interfering RNA against BNIP3 (siBNIP3)/negative control siRNA (siNC) or not, H9C2 cells were cocultured with BBR for 3 h (50 μM) or not and then exposed to H/R. **(A, B)** Western blotting to detect the protein expression of LC3, NIX, and P62; GAPDH was used as a control. **(C)** After H/R treatment, the same number of H9C2 cells from each group were cultured for 0, 12, 24, or 48 h, and cell proliferation was assessed by measuring the absorbance at 450 nm. **(D, E)** The ΔΨm of H9C2 cells was assessed by flow cytometry using R123 as a fluorescent probe after H/R, and the bar graph displays the intensity of R132 fluorescence for each group. 1, control group; 2, H/R group; 3, BBR+H/R group; 4, siNC+BBR+H/R group; 5, siBNIP3+BBR+H/R group. The data are the means ± SD (n=3). ^*^P < 0.05 compared to the control group; ^#^*P* < 0.05 compared to the H/R group; ^&^*P* < 0.05 compared to the BBR+H/R group.

Next, we used Western blotting to detect the expression levels of autophagy-associated proteins (LC3, NIX, and P62) in the different experimental groups. The results in **Figures 1A, B** show that the LC3II/I ratio and NIX expression were critically higher in H/R-treated H9C2 cells than in untreated cells, and BBR pretreatment could increase the LC3II/I ratio and NIX expression. Surprisingly, compared with siNC transfection and BBR treatment, BNIP3 knockdown significantly decreased the LC3II/I ratio and NIX expression (**Figures 1A, B**). In addition, P62 expression had the opposite trend. *In vitro*, ΔΨm and cell proliferation assays are usually used for cellular activity and damage identification, and unfavorable external stimuli often lead to an increase in ΔΨm and a decrease in cell proliferation activity, and the reduced ΔΨm usually indicate the cellular activity recovery (Zheng et al., 2017). The cell proliferation assay and ΔΨm detection showed that H/R treatment inhibited H9C2 cell proliferation and that BBR pretreatment markedly induced H9C2 cell proliferation and significantly reduced the increase in ΔΨm. ΔΨm and cell proliferation assays indicated BBR pretreatment could reduce the increase in ΔΨm due to H/R, and so protect against H/R-induced H9C2 cells apoptosis and increase H9C2 cells activity. And, after BNIP3 knockdown, BBR pretreatment did not induce H9C2 cell proliferation (**Figure 1C**) and ΔΨm decreasing (**Figures 1D, E**).

**Figure 2:**
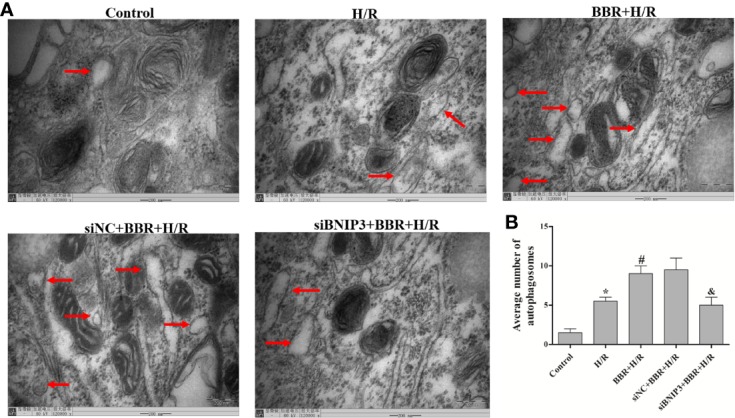
Berberine (BBR) can increase the number of autophagosomes in H9C2 cells subjected to hypoxia/reoxygenation (H/R). Autophagosomes were detected in H9C2 cells pretreated with small interfering RNA against BNIP3 (siBNIP3)/negative control siRNA (siNC) and BBR (50 μM) by transmission electron microscopy (TEM). Scale bar, 200.0 nm (insets) and magnification 120,000×. **(B)**. Quantification of the autophagosomes in different groups is presented as bar graphs. The figures are representative images of three different samples. The data are the means ± SD (n=3). ^*^P < 0.05 compared to the control group; ^#^*P* < 0.05 compared to the H/R group; ^&^*P* < 0.05 compared to the BBR+H/R group.

TEM was used to observe autophagosome formation. As illustrated in **Figure 2**, only a small number of autophagosomes were found in the control group. The number of autophagosomes increased significantly in the H/R group, and a typical bilayer membrane structure and undegraded cytoplasm were found, which shows that H/R treatment could induce autophagy. After BBR pretreatment, the number of autophagosomes increased significantly, which shows that BBR pretreatment could induce autophagy. When BNIP3 was knocked down, the effect of BBR on autophagy was weakened.

Overall, these results indicate that BBR regulates BNIP3-mediated mitochondrial autophagy to attenuate H/R injury in H9C2 cells.

### BBR Mediates BNIP3 Expression by Enhancing the Binding of HIF-1α to the BNIP3 Promoter

**Figure 3:**
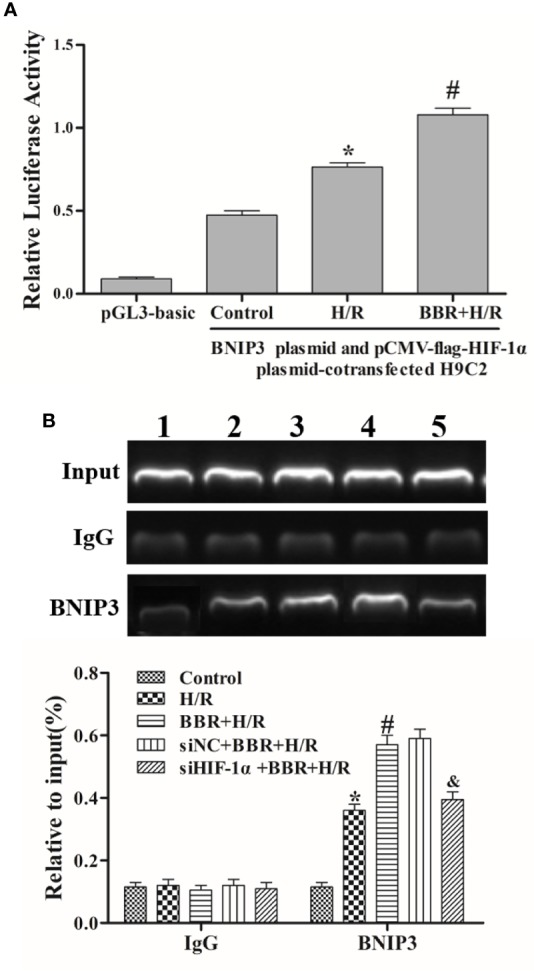
Berberine (BBR) mediates BNIP3 expression by enhancing the binding of HIF-1α to the BNIP3 promoter. **(A)** A dual-luciferase reporter assay assessed the effects of BBR on HIF-1α and BNIP3 transcriptional activity in H9C2 cells. **(B)** A chromatin immunoprecipitation (ChIP) PCR analysis was used to show the effects of BBR on HIF-1α and BNIP3 promoter binding, and HIF-1α knockdown weakened their binding activity. 1, control group; 2, H/R group; 3, BBR+H/R group; 4, siNC+BBR+H/R group; 5, siHIF-1α+BBR+H/R group. The data are the means ± SD (n=3). ^*^P < 0.05 compared to the control group; ^#^*P* < 0.05 compared to the H/R group; ^&^*P* < 0.05 compared to the BBR+H/R group.

Research has shown that the HIF-1α/BNIP3 pathway is important in hypoxia-induced autophagy in MIRI (Bruick, 2000; Tracy et al., 2007; Farrall and Whitelaw, 2009; Feng et al., 2016; Liu et al., 2019). A luciferase reporter assay was used to study the regulatory effects of BBR on HIF-1α transcriptional activity in H9C2 cells. Transfection with a reporter construct harboring the BNIP3 promoter (pGL3-BNIP3) and treatment with BBR caused a significant increase in luciferase activity compared with transfection with a reporter construct harboring the BNIP3 promoter (pGL3-BNIP3) and no BBR treatment (**Figure 3A**). These results indicate that BBR enhanced HIF-1α transcriptional activity.

Moreover, ChIP assays showed that there was no obvious binding of the HIF-1α and BNIP3 promoters under normoxia (control group), and HIF-1α and BNIP3 promoter binding significantly increased under hypoxia (H/R group). BBR further enhanced HIF-1α and BNIP3 promoter binding under H/R conditions. Additionally, when HIF-1α was silenced, the binding activity of HIF-1α and BNIP3 was weakened (**Figure 3B**), again indicating that BBR mediates BNIP3 expression by enhancing the binding of HIF-1α to the BNIP3 promoter.

### BBR Regulates BNIP3-Mediated Mitochondrial Autophagy to Attenuate I/R Injury in MIRI Model Rats

**Figure 4:**
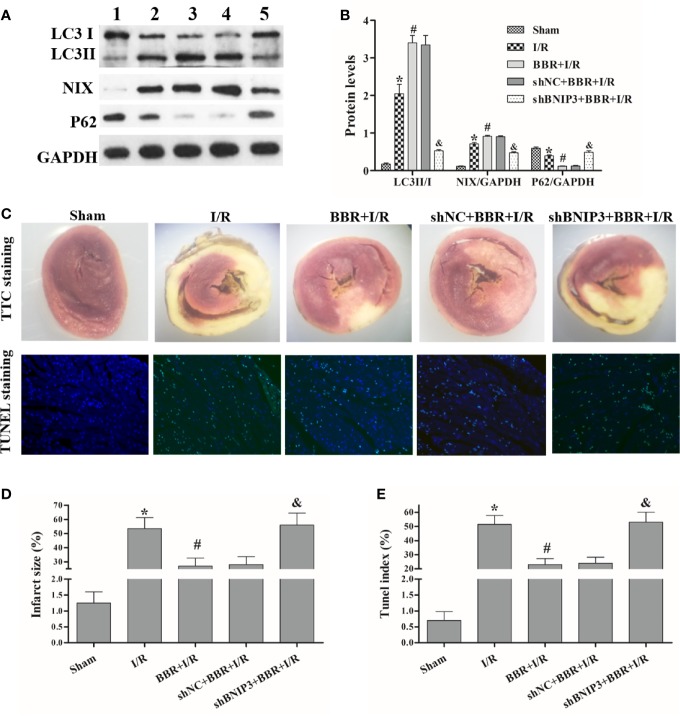
Berberine (BBR) regulates BNIP3-mediated mitochondrial autophagy to attenuate ischemia/reperfusion (I/R) injury in myocardial ischemia-reperfusion injury (MIRI) model rats. After transfection with shBNIP3/shNC, rats were administered BBR orally (300 mg/kg, once a day for 3 consecutive days) or not and then exposed to I/R. **(A, B)** Western blotting was used to detect the protein expression of LC3, NIX, and P62; GAPDH was used as a control. **(C)** After I/R, 2,3,5-triphenyl tetrazolium (TTC) staining (10×) and terminal deoxynucleotidyl transferase-mediated dUTP nick-end labeling (TUNEL) staining (400×) was performed on myocardial tissues from each group. **(D, E)** Statistical analysis of the infarct size and TUNEL index. 1, sham group; 2, I/R group; 3, BBR+I/R group; 4, shNC+BBR+ I/R group; 5,shHIF-1α+BBR+I/R group. The data are the means ± SD (n=3). ^*^P < 0.05 compared to the sham group; ^#^*P* < 0.05 compared to the I/R group; ^&^*P* < 0.05 compared to the BBR+I/R group.

In MIRI model rats, we further explored the relationship among BBR, BNIP3, and mitochondrial autophagy. Western blotting showed that the LC3II/I ratio and NIX expression were critically higher in MIRI model rats than in untreated sham rats, and BBR pretreatment could increase the LC3II/I ratio and NIX expression. Surprisingly, compared with shNC transfection and BBR treatment, BNIP3 knockdown significantly decreased the LC3II/I ratio and NIX expression (**Figures 4A, B**). In addition, P62 expression had the opposite trend.

The TTC staining results are shown in **Figures 4C, D**. The heart tissue was red in the sham group; after I/R treatment, the size of the gray/white area (that is, the myocardial infarct size) was significantly increased, but this area was smaller in BBR-treated rats. Additionally, after BNIP3 knockdown, BBR did not decrease the myocardial infarct size. These results indicate that the MIRI model was successfully established and that BBR can alleviate myocardial infarction *via* a BNIP3-mediated pathway.

**Figure 5:**
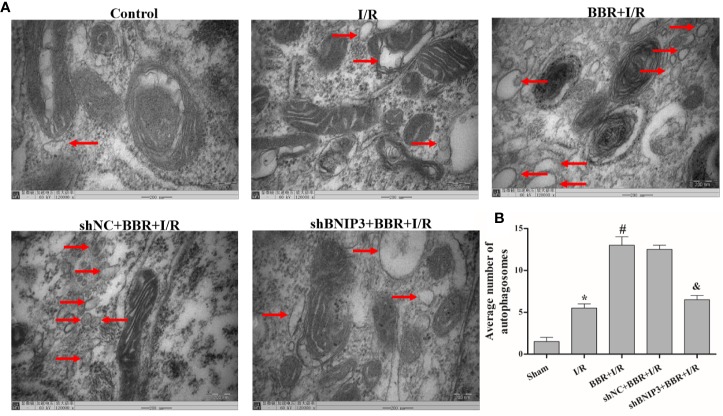
Berberine (BBR) can increase the number of autophagosomes in myocardial ischemia-reperfusion injury (MIRI) model rats subjected to ischemia/reperfusion (I/R). Autophagosomes were detected in MIRI model rats pretreated with shBNIP3/shNC and BBR (50 μM) by transmission electron microscopy (TEM). Scale bar = 200.0 nm (insets) and magnification 120,000×. **(B)**. Quantification of the autophagosomes from different groups is presented as bar graphs. The figures are representative images of three different rats. The data are the means ± SD (n=3). ^*^P < 0.05 compared to the sham group; ^#^*P* < 0.05 compared to the I/R group; ^&^*P* < 0.05 compared to the BBR+I/R group.

Next, we quantified the effect of BBR on cardiomyocyte apoptosis using a TUNEL assay. The apoptosis rates (TUNEL indexes) are shown in **Figures 4C, E**. The data show that the TUNEL indexes were greater in the I/R group than in the sham group (*P* < 0.05), and BBR treatment reduced cardiomyocyte apoptosis (TUNEL indexes). When BNIP3 was knocked down, BBR treatment did not markedly reduce cardiomyocyte apoptosis. In addtion, we also observed autophagosome formation in MIRI model rats by TEM. As shown in **Figure 5**, there were only a small number of autophagosomes in the sham group. After I/R modeling, the number of autophagosomes increased significantly in the I/R group, which shows that I/R modeling could induce autophagy. After BBR pretreatment, the number of autophagosomes increased significantly, which shows that BBR pretreatment could also induce autophagy. However, after BNIP3 gene was knocked down (shBBIP3), the number of autophagosomes decreased significantly, which shows that the effect of BBR on autophagy was weakened.

Moreover, BBR could regulate myocardial enzyme activity. As shown in **Table 1**, CK-MB, LDH and AST activity was significantly higher in the I/R group than in the sham group (*P* < 0.05). BBR pretreatment markedly decreased CK-MB, LDH and AST activity-induced I/R. There were no significant changes in myocardial enzyme activity between the shBNIP3+BBR+I/R group and the I/R group, thus indicating that BBR could not effectively regulate myocardial enzyme activity when BNIP3 was silenced.

**Table 1:** CK-MB, LDH, and AST concentrations in each group (n = 5).

	Sham	I/R	BBR+I/R	shNC+BBR+I/R	shBNIP3+BBR+I/R
CK-MB (U/L)	57.8 ± 4.2	92.8 ± 3.27^*^	78.2 ± 7.2^#^	76.6 ± 7.5	94.0 ± 8.0^&^
LDH (U/L)	280.0 ± 14.4	475.6 ± 14.5^*^	356.6 ± 18.4^#^	367.6 ± 26.9	480.2 ± 17.6^&^
AST (U/L)	32.4 ± 6.4	83.4 ± 7.6^*^	52.6 ± 8.2^#^	49.6 ± 8.5	82.2 ± 7.7^&^

^*^P < 0.05 compared to the sham group; ^#^P < 0.05 compared to the I/R group; ^&^P < 0.05 compared to the BBR+I/R group.

These results show that BBR reduces I/R-induced cardiomyocyte apoptosis and alleviates MIRI injury *via* regulating BNIP3-mediated mitochondrial autophagy.

## Discussion

As a natural substance, BBR has protective effects on MIRI and has attracted the attention of researchers (Huang et al., 2015; Wang et al., 2015; Zhang et al., 2016). However, the clinical application of BBR still lacks scientific basis, and the protective mechanisms of BBR against MIRI are still not well known. In this study, we found that BBR could markedly induce H9C2 cells proliferation and inhibit apoptosis (ΔΨm decrease) *in vitro* experiment, and alleviate myocardial infarction area and cardiomyocytes apoptosis and reduce myocardial enzyme activity (AST, CK-MB and LDH) *in vivo* model. Importantly, we verified for the first time that BBR induce mitophagy *via* the HIF-1α/BNIP3 pathway. Based on these studies, we believe that BBR protects the heart and has beneficial effects on MIRI, and may serve as a novel therapeutic drug for myocardial I/R injury.

The autophagy process is controlled by autophagy-related genes, which are largely involved in autophagosome formation. The conversion of the soluble form of LC3 (LC3-I) to the autophagic vesicle-associated form (LC3-II) is considered to be a major marker of autophagy (Nishida et al., 2008). NIX (also known as BNIP3L) is a Bcl-2-related protein, and BNIP3 is involved in mitochondria removal during the autophagic response; hypoxia-induced autophagy depends on both BNIP3 and NIX (Zhang et al., 2009; Bellot et al., 2009). P62, a selective substrate for autophagy, is a preferred target for autophagy and directly interacts with LC3 (Ichimura et al., 2008). Autophagy is accompanied by changes in autophagy-related protein expression levels and an increase in the number of autophagosomes (Kimura et al., 2007). TEM is the standard method for observing autophagosome formation. Using Western blotting and TEM, we found that the LC3II/I ratio and NIX expression levels increased, P62 expression levels decreased, and the autophagosome number increased after BBR pretreatment. These data show that BBR improves MIRI by promoting autophagy levels.

Autophagy is a dynamic catabolic process in which basal autophagy is usually induced for material re-use and body protection through protein and organelle turnover and recycling. However, when cells are exposed to unfavorable stimuli such as hypoxia, nutrient deficiency or radiation, autophagy is rapidly activated or upregulated as an adaptive response (Levine and Kroemer, 2008). Mounting evidence has revealed that autophagy is involved in a wide range of physiological processes and the pathogenesis of a variety of diseases, such as MIRI, acute lung injury, and various types of infections (Hamacherbrady et al., 2007; Dong et al., 2010; Yang et al., 2010; Sun et al., 2012; Li et al., 2016; Wu et al., 2018). As a pivotal mediator of the hypoxic signal responsible for the transcription of hundreds of downstream target genes, HIF-1a has been implicated in hypoxia-induced autophagy (Zhao et al., 2012). BNIP3, a BH3-only Bcl-2 family member known as pro-apoptotic protein, can induce apoptotic responses and mitochondrial autophagy (Zhang et al., 2008). During hypoxia-induced autophagy, BNIP3 expression is induced through HIF-1α binding to the BNIP3 promoter, which means that HIF-1a can induce BNIP3 expression (Bruick, 2000). Under hypoxic conditions, a ChIP assay showed that the binding of HIF-1α to the BNIP3 promoter was significantly enhanced (Tracy et al., 2007; Farrall and Whitelaw, 2009). Research has shown that the HIF-1α/BNIP3 pathway is important in hypoxia-induced autophagy in MIRI. For example, the protective effect of Panax notoginseng saponins on MIRI was due mainly to its ability to enhance mitochondrial autophagy in myocardial tissue through the HIF-1α/BNIP3 pathway (Liu et al., 2019). Prolonged hypoxia-induced HIF-1α stimulated BNIP3 activation to regulate mitochondria-dependent cardiomyocyte apoptosis by inhibiting the IGF1R/PI3K/Akt survival pathway (Feng et al., 2016). In the present study, luciferase reporter and ChIP assays indicated that under H/R conditions, BBR mediated BNIP3 expression by enhancing the binding of HIF-1α to the BNIP3 promoter. Furthermore, BBR pretreatment aggravated mitochondrial autophagy (autophagy-related proteins changed, and the autophagosome number increased), but inhibiting BNIP3 expression (siBNIP3 and shBNIP3 transfection) attenuated BBR-induced mitochondrial autophagy. Therefore, we conclude that BBR may regulate mitochondrial autophagy *via* the HIF-1α/BNIP3 pathway.

**Figure 6:**
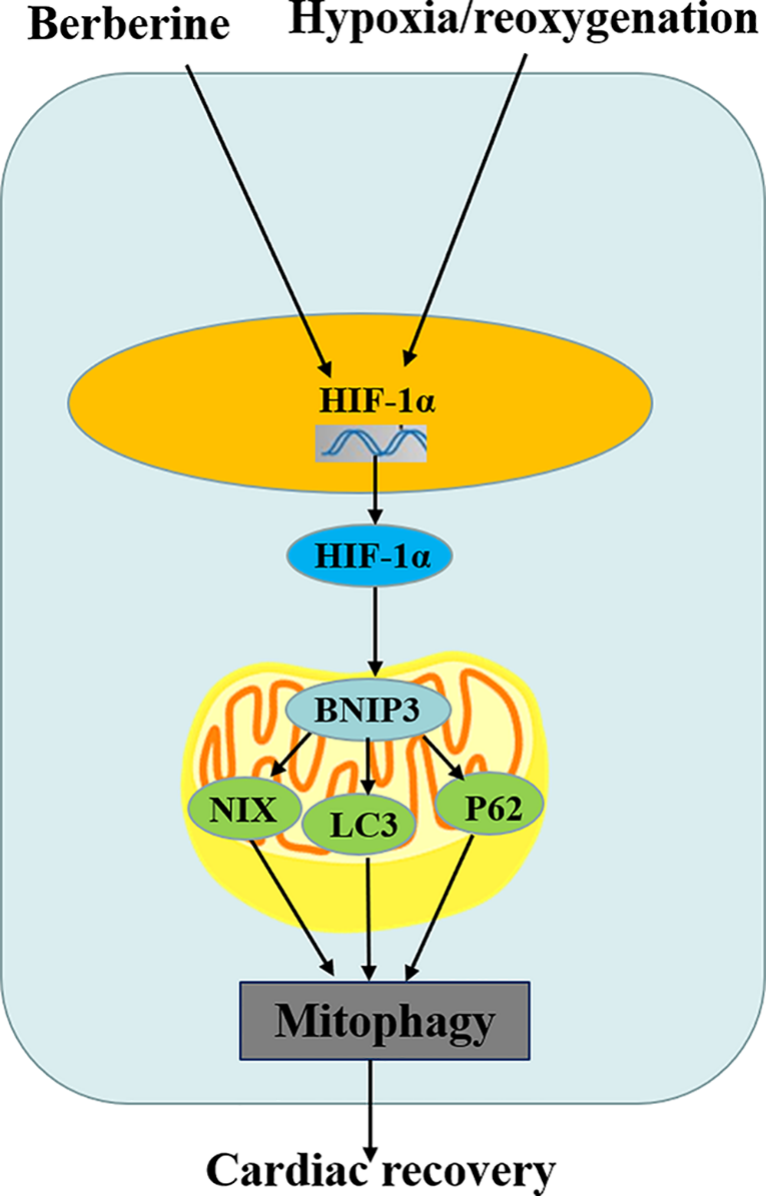
Schematic diagram of berberine (BBR) regulating cardiomyocytes mitophagy.

Researchers have found that a variety of natural or chemical substances have protective effects against MIRI. Shibata et al. indicated that in ischemia-reperfusion mice, adiponectin could decrease myocardial infarct size, reduce myocardial apoptosis and TNF-α expression, and protect the hearts from I/R injury through both AMPK- and COX-2-dependent mechanisms (Shibata et al., 2005). Ji et al. found that genistein reduced myocardial infarct size and myocardial apoptosis in I/R rabbits (Ji et al., 2004). Ito et al. noted that pioglitazone reduced the number of infiltrating macrophages in the ischemic region and had antiinflammatory effects against MIRI (Ito et al., 2004). As a natural substance of traditional Chinese medicine, numerous clinical and experimental studies have confirmed that BBR has protective effects on MIRI (Huang et al., 2015; Wang et al., 2015; Zhang et al., 2016). In this study, we found that BBR could inhibit cardiomyocyte apoptosis and induce mitophagy *via* the HIF-1α/BNIP3 pathway (**Figure 6**), but it still needs further in-depth study whether BBR has other protective mechanisms just like the natural or chemical substances above, such as antiinflammatory activity.

## Conclusion

In conclusion, we found that BBR could promote mitochondrial autophagy, reduce myocardial enzyme activity, induce cardiomyocytes proliferation, inhibit cardiomyocytes apoptosis, and protect the heart from myocardial I/R injury, possibly through the HIF-1α/BNIP3 pathway. Based on these studies, we believe that BBR may serve as a novel therapeutic drug for myocardial I/R injury.

## Data Availability Statement

All datasets generated for this study are included in the article/**Supplementary Materials**.

## Ethics Statement

The animal study was reviewed and approved by the Institutional Animal Care and Use Committee of Henan Provincial People's Hospital.

## Author Contributions

Conceived and designed the experiments: XC, JL, and LL. Performed the experiments: XC, NZ, YL, and YZ. Collected the samples: QD and PH. Analyzed the data: JYL. Wrote the paper: XC and NZ.

## Funding

This study was supported by grants from the National Natural Science Foundation of China (project number: U1704167, to XC), the Henan Provincial Science and Technology Project (project number: 182102310525, to XC; 202102310088, to JL and 172102310063, to PH), the Joint Construction Project of Henan Medical Science and Technology Tackling Plan (project number: 2018020475, to NZ), Henan Medical Science and Technology Tackling Plan Provincial-ministerial Co-construction Project (project number: SB201902033, to XC), and Key Scientific Research Projects of Colleges and Universities of Henan Province (project number: 20A320086).

## Conflict of Interest

The authors declare that the research was conducted in the absence of any commercial or financial relationships that could be construed as a potential conflict of interest.
