# Supplementary material for: Berberine Protects Against Simulated Ischemia/Reperfusion Injury-Induced H9C2 Cardiomyocytes Apoptosis In Vitro and Myocardial Ischemia/Reperfusion-Induced Apoptosis In Vivo by Regulating the Mitophagy-Mediated HIF-1α/BNIP3 Pathway
Source: Front Pharmacol. 2020 Mar 27;11:367. doi: 10.3389/fphar.2020.00367 (PMC7120539; doi:10.3389/fphar.2020.00367)

Supplemental Figure 2. BBR regulates BNIP3 expression in MIRI model rats. After transfection with shBNIP3/shNC, rats were administered BBR orally (300 mg/kg, once a day for 3 consecutive days) and then exposed to I/R. (A and B) RT-PCR and Western blotting were used to detect the mRNA and protein expression of BNIP3. GAPDH was used as a control. (C and D) RT-PCR and Western blotting were used to detect the mRNA and protein expression of BNIP3 after shBNIP3 or shNC transfection. The data are the means ± SD (n=3). $^{*}P < 0.05$ compared to the sham group; $^{\#}P < 0.05$ compared to the I/R group; $^{\&}P < 0.05$ compared to the shNC group.

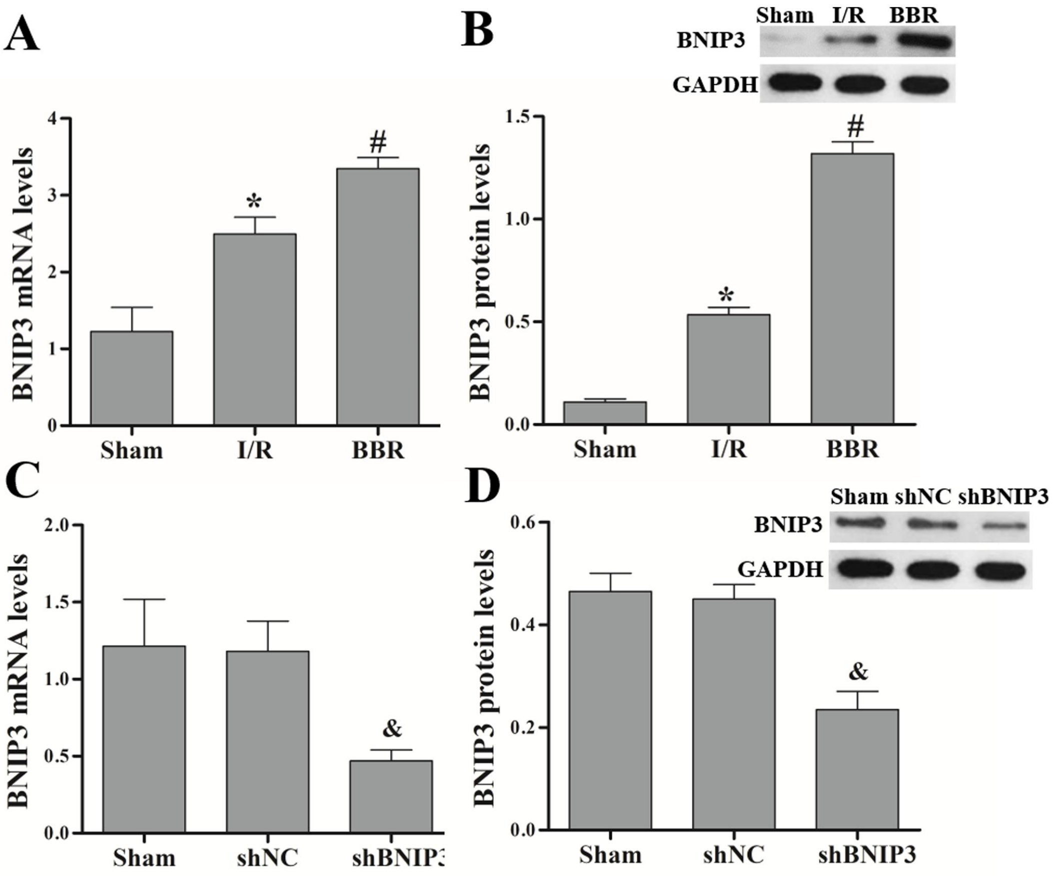

Supplement: Supplementary file 2 [file Image_2.pdf]
