# Supplementary material for: Berberine Protects Against Simulated Ischemia/Reperfusion Injury-Induced H9C2 Cardiomyocytes Apoptosis In Vitro and Myocardial Ischemia/Reperfusion-Induced Apoptosis In Vivo by Regulating the Mitophagy-Mediated HIF-1α/BNIP3 Pathway
Source: Front Pharmacol. 2020 Mar 27;11:367. doi: 10.3389/fphar.2020.00367 (PMC7120539; doi:10.3389/fphar.2020.00367)
Supplement: Supplementary file 3 [file Table_1.doc]

**Supplemental Table 1.** Primers used for cell transfection and Luciferase reporter assay.

| Gene | Name | Primer (5’-3’) |
| --- | --- | --- |
| siBNIP3 | siBNIP3-sense | UAAGUACAAACCGUAGAACUU |
|  | siBNIP3-antisense | GUUCUACGGUUUGUACUUAAA |
| siHIF-1α | siHIF-1α-sense | AAAACUUCAGACUCUUUGCUU |
|  | siHIF-1α-antisense | GCAAAGAGUCUGAAGUUUUUU |
| siNC | siRNA control-sense | CCAAAGCGGTTGACCCATT |
|  | siRNA control-antisense | AATGGGTCAACCGCTTTGG |
| BNIP3 | BNIP3-Luc-f | atctgcgatctaagtaagcttTCCACCTGCCTCTGACACCA |
|  | BNIP3-Luc-R | tccagcggatagaatggcgccAGGGGCGGGGCGGGGCGG |
