# Supplementary material for: Berberine Protects Against Simulated Ischemia/Reperfusion Injury-Induced H9C2 Cardiomyocytes Apoptosis In Vitro and Myocardial Ischemia/Reperfusion-Induced Apoptosis In Vivo by Regulating the Mitophagy-Mediated HIF-1α/BNIP3 Pathway
Source: Front Pharmacol. 2020 Mar 27;11:367. doi: 10.3389/fphar.2020.00367 (PMC7120539; doi:10.3389/fphar.2020.00367)
Supplement: Supplementary file 4 [file Table_2.doc]

**Supplemental Table 2.** Primers used for quantitative real-time PCR analysis.

| Primer | Forward (5’-3’) | Reverse (5’-3’) |
| --- | --- | --- |
| BNIP3 | ATTGGTCAAGTCGGCCAGAA | TCATGCTGAGAGTAGCTGTGC |
| GAPDH | TGACAACTTTGGCATCGTGG | GGGCCATCCACAGTCTTCTG |
